# Prospective clinical evaluation of 765 partial glass-ceramic posterior restorations luted using photo-polymerized resin composite in conjunction with immediate dentin sealing

**DOI:** 10.1007/s00784-020-03454-7

**Published:** 2020-08-12

**Authors:** Carline R. G Van den Breemer, Gerrit J. Buijs, Marco S. Cune, Mutlu Özcan, Wouter Kerdijk, Stephan Van der Made, Marco M. M. Gresnigt

**Affiliations:** 1 Department of Restorative Dentistry and Biomaterials, Center for Dentistry and Oral Hygiene, University Medical Center Groningen, Antonius Deusinglaan 1, 9713 AV Groningen, The Netherlands; 2 Groningen, The Netherlands; 3 Department of Oral-Maxillofacial Surgery, Prosthodontics and Special Dental Care, St. Antonius Hospital Nieuwegein, Nieuwegein, The Netherlands; 4 Department of Oral Maxillofacial Surgery, Prosthodontics and Special Dental Care, University Medical Center Utrecht, Utrecht, The Netherlands; 5 Division of Dental Materials, Center for Dental and Oral Medicine, Clinic for Fixed and Removable Prosthodontics and Dental Materials Science, University of Zurich, Zurich, Switzerland; 6 Department of Education and Research, Hanze University of Applied Sciences, Groningen, The Netherlands; 7 Kwalident Dental Studio, Beilen, The Netherlands; 8 Department of Special Care, Martini Hospital, Groningen, The Netherlands

**Keywords:** Adhesion, Posterior, Immediate dentin sealing, Partial restorations, Lithium disilicate, Survival

## Abstract

**Objectives:**

To evaluate the clinical performance of partial glass-ceramic (IPS e.max Press) posterior restorations.

**Materials and methods:**

A total of 765 restorations in 158 patients were placed between 2008 and 2018 and evaluated in a prospective study during regular dental care visits between 2015 and 2018. The restorations were luted with a conventional photo-polymerized resin composite (HFO) in conjunction with an Immediate Dentin Sealing procedure (IDS). Intra-oral photographs and radiographs were made and evaluated using USPHS criteria.

**Results:**

The mean observation time was 53.3 months (range 3–113 months). Three absolute failures occurred (tooth fractures, *n* = 2; apical re-infection, *n* = 1) all leading to the loss of the restored tooth. Repairable and salvageable failures occurred in 9 teeth (endodontic complications, *n* = 7; secondary caries, *n* = 1; debonding, *n* = 1). The survival and success rates according to Kaplan-Meier after 5 years cumulated to 99.6% and 98.6%, respectively. Location (premolar/molar and mandibula/maxilla), pre-restorative endodontic status (vital/devitalised) and extension of the indirect ceramic restoration (number of sides and cusps involved) did not significantly affect the cumulative success rate (log rank test, *p* > 0.05). The condition of the vast majority of the restorations remained unaffected for 5 years.

**Conclusions:**

Partial glass-ceramic posterior restorations (pressed lithium disilicate (IPS e.max press, Ivoclar Vivadent) luted by means of a conventional photo-polymerized resin composite in conjunction with the use of an IDS procedure have an excellent medium-term prognosis.

**Clinical relevance:**

Partial glass-ceramic posterior restorations can be considered as a highly reliable treatment option. Location and extension of the restoration and pre-restorative endodontic status do not affect success rate.

## Introduction

Partial indirect restorations can be indicated when it is difficult to restore form and function using direct composite restorations. Monolithic reinforced glass-ceramic restorations have gained popularity in posterior teeth as these restorations are less prone to fracture compared to feldspathic ceramic [1]. Lithium disilicate restorations have an increased fracture toughness from the crystallites as these induce the fracture to bow, deflect and branch [2]. Besides their strength, adhesive luting is possible and this increases strength and regain aesthetics at a minimum biological price, removing defect structures only [3–5].

A recent systematic literature review revealed a cumulative survival rate for single glass-ceramics and feldspathic porcelain restorations of 92–95% after 5 years, and 91% after 10 years [6]. Due to high load in the posterior region, these restorations were more prone to fracture than crowns in the anterior region [7, 8]. In short-term evaluations, bulk fracture, chipping of the ceramic and adhesive problems have been reported as the main reason of failure [9]. Besides restoration failures, secondary caries was observed as a biological complication (1%) [6]. Comparing acid-etched e.max lithium disilicate monolithic and bilayered complete coverage restorations resulted in an estimated cumulative survival rate of 96.5% after 10.4 years for monolithic and 100% after 7.9 years for bilayered restorations. This difference was statistically significant [10]. Long-term data with a high number of these partial all-ceramic restorations is unfortunately limited, which precludes firm statements regarding their effectiveness [11].

Adhesive bonding to dentin has been considered as the weakest link in clinical durability and fracture resistance of ceramic restorations [12]. The so-called immediate dentin sealing concept (IDS) has been studied extensively in in vitro studies and obtained significantly increased bond strengths [13–21]. An vitro study demonstrated that the application of an IDS layer with ceramic inlays significantly increased fracture strengths [22].

The use of a conventional photo-polymerized resin composite as a luting agent is debated [23, 24]. Manufacturers and studies claimed that the use of composite as a luting agent with thick or extensive restorations could lead to a decrease in degree of conversion [25–29]. However, luting with composite has some practical advantages, i.e., increased working time, improved biomechanical properties, wear resistance and ease of removal the excess [25–27, 30]. In addition, recent articles have proved their ability to have a higher bond strength in in vitro [26, 31] and in in vivo research [27, 30, 32].

The aim of this study is to evaluate the clinical survival and success rate of partial glass-ceramic posterior restorations luted with conventional photo-polymerized resin composite in conjunction with the use of IDS. Further evaluation will involve the location of the restoration (premolar/molar and mandibula/maxilla), pre-restorative endodontic status (vital/devitalised) and the extension of the indirect ceramic restoration (number of sides and cusps involved).

## Materials and methods

### Study design and inclusion

All patients, who received posterior partial lithium disilicate ceramic restorations between 2008 up to 2018, were eligible for inclusion for this prospective study. Patients were treated by one operator in a private practice, and all restorations were luted with a conventional photo-polymerized resin composite in conjunction with an IDS. Indirect restorations were provided for various reasons: secondary caries, replacement of a failing direct restoration or fracture of one of the cusps. To be eligible, patients should not have active periodontal or pulpal diseases. This study was evaluated by the medical ethical board of the University Medical Center Groningen and registered in the clinical trial register (NCT3452358). The product, manufacturers and chemical compositions of the materials that were used in this study are listed in Table 1.

**Table 1:** The products, manufacturers and chemical composition of the material used in this study

Product	Composition
IPS e.max Press	Pressable ceramic
(Ivoclar Vivadent, Schaan, Liechtenstein)	
HFO – composite enamel plus UD2	1,4-Butandioldimethacrylate, urethane
(Micerium S.p.A., Avegno, Italy)	dimethacrylate, Bis-GMA
CoJet®-sand	Aluminium trioxide particles coated with silica, 30 μm
(3M ESPE, Neuss, Germany)	
ESPE®-sil	Ethyl alcohol, 3-methacryloxy-propyltrimethoxysilane, ethanol
(3M ESPE, Neuss, Germany)	
Monobond Plus	One component primer
(Ivoclar Vivadent, Schaan, Liechtenstein)	
Clearfil SE Bond	Primer: HEMA, hydrophilic dimethacrylate, water, photo initiator
(Kuraray, Osaka, Japan)	
	Adhesive: TEDGMA, UDMA, GPDM, HEMA, bis-GMA, filler, photo initiator
Porcelain etch	9% Hydrofluoric acid
(Ultradent, South Jordan, UT, USA)	
Ultra-etch	35% Phosphoric acid
(Ultradent, South Jordan, UT, USA)	
GrandIO flow	1,6-Hexanediylbismethacrylate, BIS-GMA, triethylene glycol dimethacrylate
(VOCO, Cuxhaven, Germany)	
K-Y* lubricating gelly	Purified water, glycerin, methylparaben, propylparaben, propylene glycol, hydroxyethylcellulose, dissodium, phosphate, sodium phosphate, tetrasodium, EDTA
(Johnson & Johnson, Sezanne, France)	
Hygenic Dental dam	Rubberdam
(Coltène/Whaledent Inc., Ohio, USA)	
Nic tone Dental Dam	Rubberdam
(MDC Dental, Zapopan, Jalisco, Mexico)	
Durelon	Powder: zinc oxide, stannous fluoride, tin dioxide Liquid: water, polyacrylic acid
(3M ESPE, Neuss, Germany)	
Brownies	Polisher, medium
(Shofu, Kyoto, Japan)	
Impression material	Hydrocolloid impression material
(VanR Heavy bodied, DUX Dental, USA)	

### Tooth preparation

All procedures were performed using high magnification × 8–25 (OpmiPico, Zeiss, Jena, Germany). After isolation using a rubberdam (Hygenic Dental Dam, Coltène Whaledent Inc., Ohio, USA and Nic tone, MDC Dental, Zapopan, Jalisco, Mexico), the existing restorative material was removed. Minimal invasive preparations were made, and sound enamel was not removed. Outlines consisted of a shoulder or chamfer made with a red handpiece and diamond burs (type: 881G 012, 014 and 016; 880G 023, 881F 012, 8881 314 014, 016 (Meisinger, Neuss, Germany)) or with a SONICflex prep ceram handpiece (KaVo, Biberach/Riß, Germany). All internal angles or undercuts were not smoothened but covered using IDS with flowable composite. Regardless of the endodontic status, the cusps were only covered if they were already part of the old restoration or when a fracture line was visible in > 50% of the cusp. The proximal walls were slightly diverging with an angle of 100° to 120° between the proximal cavity walls and the prospective proximal inlay surface. Occlusal marginal ridge contacts were not avoided. Occlusal thickness of the restoration after removing the old restorations was at least 0.5 mm [33–35]. Immediately following preparation, the tooth received IDS (Clearfil SE Bond, Kuraray, Osaka, Japan). The clinical protocol for tooth preparation and IDS is presented in detail in Table 2. To obtain a smooth surface and to compensate for incidental undercuts after preparation, a flowable resin was applied (GrandIO flow, VOCO, Cuxhaven, Germany). Electrosurgery was performed in cases where retraction of the gingiva was needed to obtain a proper and detailed impression. Impressions were made using a hydrocolloid impression material (VanR Heavy bodied, DUX Dental, USA). Temporary restorations were made chair-side using a chemical cured provisional material (Protemp, 3M ESPE, Seefeld, Germany). They were temporarily luted using a polycarboxylate cement (Durelon, 3M ESPE Seefeld, Germany).

**Table 2:** Clinical protocol for tooth preparation and Immediate Dentin Sealing

First visit: Preparation of the tooth before impression *Tooth preparation is ready for impression, followed by:	
1.1 Apply SE Primer (Clearfil SE Bond, Kuraray), active brushing motion	20 s
1.2 Air suction	
1.3 Apply SE Adhesive (Clearfil SE Bond, Kuraray), active brushing motion	10 s
1.4 Air-thin	10 s
1.5 Photo-polymerize	10 s
1.6 Apply flowable resin (GrandIO flow, VOCO, Cuxhaven, Germany)	
1.7 Photo-polymerize	40 s
1.8 Apply glycerin gel (K-Y* lubricating gelly, Johnson & Johnson, Sezanne, France)	
1.9 Photo-polymerize at buccal, oral and proximal sites	40 s each
1.10 Rinse until clean surface	
1.11 Clean enamel outline with a rubberpoint or a bur	
1.12 Take impression	
Second visit: Preparation of the tooth before luting	
2.1 Clean tooth surface ultrasonically or with a scaler	
2.2 Silica coat the immediate dentin sealing layer (CoJet®-sand, 3 M ESPE, Neuss, Germany)	2–3 s
2.3 Acid etch the enamel (not the accidently exposed dentin)	30 s
2.4 Rinse	30 s
2.5 Dry	
2.6 Apply Silane (ESPE – Sil, 3 M ESPE) on the immediate dentin sealing layer	60 s
2.7 Apply SE Adhesive (Clearfil SE Bond, Kuraray)	10 s
2.8 Apply composite (HFO composite, Micerium S.p.A., Avegno, Italy) onto the tooth	
2.9 Place the partial restoration onto the tooth	
2.10 Remove excess of cement	
2.11 Photo-polymerize	40 s
2.12 Apply of glycerin gel (K-Y* lubricating gelly, Johnson & Johnson)	
2.13 Photo-polymerize at buccal, oral and proximal sides	40 s each

### Luting procedure

All pressed lithium disilicate restorations (IPS e.max press, Ivoclar Vivadent) were fabricated in a dental laboratory using magnification loups × 4.2 (Examvision, Rotterdam, The Netherlands) and microscope × 8–25 (OpmiPico, Zeiss) following the manufacturer’s instructions. Restorations were made by the lost wax technique using pressable ceramics (IPS e.max, Ivoclar Vivadent). To get correct shade integration, a staining technique was used (IPS e.max Stains, Ivoclar Vivadent) and glazed afterwards (IPS e.max Fluoglaze, Ivoclar Vivadent). Hereafter, the restorations were handpolished (Signum HP diamond polishing, Hereaus Kulzer GmbH, Hanau, Germany). The clinical protocol for preparation of tooth and the ceramic restorations are presented in detail in Tables 2 and 3. The temporary restoration was removed, and the teeth were cleansed from temporary cement with an ultrasonic tip or a hand scaler. The IDS layer was silica coated (30 μm SiO_2_ Cojet-sand, 3 M ESPE) using an intra-oral air-abrasion device (Dento-prepTM, RØNVIG A/S, Daugaard, Denmark) at a pressure of 2.5 bar from a distance of approximately 10 mm for 2–3 s. The adjacent teeth were protected using a metal strip during air-abrasion procedure. Try-in of the partial ceramic restoration was done, and margins were checked. Subsequently, the adjacent teeth were protected with teflon tape (PTFE tape) and enamel etched with phosphoric acid (Ultradent, South Jordan, UT, USA). Then, the preparation was rinsed with copious water for 20 s, dried with oil-free compressed air and silane (EPSE-sil, 3M ESPE, Neuss, Germany) applied at the IDS layer and left to react with the silica particles for 1 min.

**Table 3:** Clinical protocol for luting procedures of the ceramic restorations

1. Etch ceramic with hydrofluoric acid (IPS Ceramic etch, Ivoclar Vivadent)	20 s
2. Rinse with neutralized water (Neutralizing powder, Ivoclar Vivadent)	60 s
3. Dry	
4. Clean ceramic ultrasonically in distilled water	300 s
5. Dry	
6. Apply of silane (Monobond Plus, Ivoclar Vivadent)	60 s
7. Apply SE Adhesive (Clearfil SE Bond, Kuraray)	10 s

*For the following procedures see step 2.8 Table 2

After etching the restoration with 9% hydrofluoric acid (Porcelain etch, Ultradent), the restorations were ultrasonically cleaned in distilled water for 5 min. After cleaning, the intaglio surface of the lithium disilicate restoration was silanized (Monobond Plus, Ivoclar Vivadent). The procedure for conditioning the restorations prior to luting is described in detail in Table 3. All partial restorations were luted using a heated (55 °C; RØNVIG A/S) photo-polymerized resin composite (HFO composite, Micerium S.p.A., Avegno, Italy). After placement under soft pressure, excess cement at the margins was removed with a dental probe. After applying increasing pressure the additional excess cement was manipulated against the tooth with a probe and brush in order to prevent marginal gaps. The restorations were photo-polymerized for 3 times 40 s from all 3 sides. This was repeated after application of glycerin gel to ensure oxygen inhibition during polymerization. Excess composite was removed after rinsing the glycerin gel, with a scaler and an EVA-handpiece (7LP in combination with a 61 LG) (Kavo, Biberach/Riß, Germany). Final polishing was performed with a brownie (Shofu, Kyoto, Japan).

At the time of placement (baseline), the restorations and clinical circumstances were fully documented, including intra-oral photographs and radiographs. The location of the restoration (premolar/molar and mandibula/maxilla), pre-restorative endodontic status (vital/devitalised) and the extension of the indirect ceramic restoration (number of sides (buccal/lingual/palatinal/mesial/distal) and cusps involved) was noted.

### Evaluation

All patients were evaluated at regular intervals (i.e., every 6 months), with special emphasis and attention paid to the partial restoration(s) every time they visited the clinic for regular dental check-ups between 2015 and 2018.

To assess the condition of the restorations, a light photograph was made with a digital camera (Nikon (D7100, 60-mm lens), Nikon, Amsterdam, The Netherlands) at each follow-up session and subsequently evaluated by an independent researcher according to the modified United States Public Health Service (USPHS) criteria (Table 4, 11 parameters). Intra-oral radiographs were made when indicated. Patient’s records were checked for the occurrence of failures. Restorations were not replaced after endodontic treatments but restored by a composite restoration and continued to follow-up. The findings were to be compared with those obtained at baseline and to all other follow-up events.

**Table 4:** Criteria used for the clinical evaluations of the restorations (adapted version of modified United States Public Health Service (USPHS) criteria)

Category	Score*	Criteria
1. Photograph—adaptation restoration	0	Restorations contour is continuous with existing anatomical form and margins of the restoration
	1	Restoration is slightly under of over contoured
	2	Marginal overhang or tooth structure (dentin or enamel) is exposed
	3	Restoration is missing, traumatic occlusion or restoration cause pain in tooth or adjacent tissue
2. Photograph—caries	0	No visible caries
	1	Caries contiguous with the margin of the restoration
3. Photograph—marginal adaptation	0	Excellent continuity at resin—enamel interface; no ledge formation, no discoloration
	1	Slight discoloration at resin—enamel interface; ledge at interface
	2	Moderate discoloration at resin—enamel interface measuring 1 mm or greater
	3	Recurrent decay at margin
4. Photograph—polishability	0	Smooth and highly shiny, similar to enamel
	1	Smooth and satin, highly reflective
	2	Rough and shiny, satin, somewhat reflective
	3	Rough and dull or satin, not reflective
5. Photograph—surface staining	0	Absent
	1	Present
6. Photograph—contact points	0	Present
	1	Absent
7. Photograph—fracture of restoration	0	No fracture of the restoration
	1	Small lines of the restoration
	2	Small chippings (1/4 of restoration)
	3	Moderate chippings (1/2 of restoration)
	4	Severe chippings (3/4 of restoration)
	5	Loose of the restoration
8. Photograph—wear restoration	0	No wear
	1	Wear
9. Radiopgraph—adaptation restoration	0	Restorations contour is continuous with existing anatomical form and margins
	1	Restoration is slightly under of over contoured
	2	Marginal overhang or tooth structure (dentin or enamel) is exposed
	3	Restoration is missing, traumatic occlusion or restoration cause pain in tooth or adjacent tissue
10. Radiopgraph—caries	0	No visible caries
	1	Caries contiguous with the margin of the restoration
11. Radiopgraph—marginal adaptation	0	Excellent continuity at resin—enamel interface; no ledge formation, no discoloration
	1	Slight discoloration at resin—enamel interface; ledge at interface
	2	Moderate discoloration at resin—enamel interface measuring 1 mm or greater
	3	Recurrent decay at margin

*Scores 0, 1, 2, 3, 4 and 5 can also be read as Alpha, Beta, Charlie, Delta, Echo and Foxtrot

The data for all 11 USPHS parameters were presented at yearly intervals, with observation periods stretching from ‘baseline’ to ‘5 years and longer’. The observations were attributed to the closest full year.

### Statistical analysis

For an estimation of the cumulative success and survival rate in relation to observation time, the Kaplan-Meier method was used. Subgroup analysis for the cumulative survival and success rate was performed for location (mandibula/maxilla), pre-restorative endodontic status (vital/devitalised) and the extension of the indirect ceramic restoration (number of surfaces and cusps involved) using the log rank test.

Only restorations with ratings of 0 in the USPHS criteria were considered a ‘success’; hence, for the success rate, ‘an event’ was defined when a USPHS-score larger than 0 was observed on any of the 11 evaluated qualitative aspects. For the calculation of the chance on survival, ‘an event’ was noted when the restoration or the tooth itself was no longer salvageable.

The USPHS scores for the various domains are tabulated across 6 observation periods using descriptive statistics. A USPHS sum score is calculated as a general indication for the quality of the restoration, by adding the scores for the various domains. Mean values and standard deviations are calculated and compared in time using non parametric statistics (Kruskal-Wallis test and post hoc Mann-Whitney *U* tests, after Bonferroni correction *p* values < 0.0033 were considered statistically significant.

Statistical analyses were performed with a statistical software program (SPSS 22.0; SPSS Inc., Chicago, IL, USA).

## Results

In total, 765 partial restorations in 158 patients (60 men, 98 women) could be included: of which 697 restorations on vital teeth and 68 on devitalised teeth. In total, 360 restorations were placed in the mandibula and 405 in the maxillary posterior teeth, 282 on premolars and 483 on molars. The mean observation time was 53.3 months (range 3–113 months).

In three cases, the extension of the indirect ceramic restoration could not be determined from the photograph made at baseline, nor from the patients’ record. The number of sides (buccal/lingual/palatinal/mesial/distal) that the restorations replaced were as follows: 2 sides: 12, 3 sides: 198, 4 sides: 262 and 5 sides: 290. The number of cusps that were replaced by the restoration were as follows: 0 cusp *n* = 409, 1 cusp *n* = 179, 2 cusp *n* = 110, 3 cusps *n* = 19 and 4 cusps *n* = 45.

**Fig. 1:**
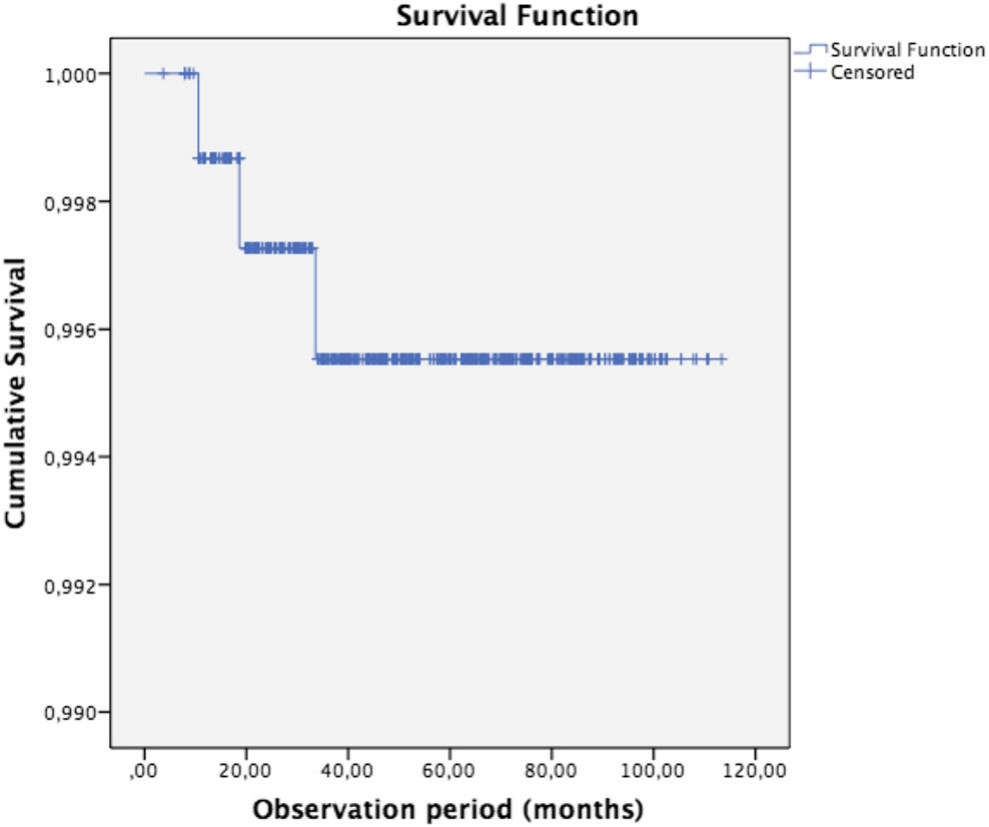
Kaplan-Meier curve of the cumulative survival rate after 5 years and longer is 99.6% SE 0.3% (*n* = 765 at baseline, events *n* = 3)

**Fig. 2:**
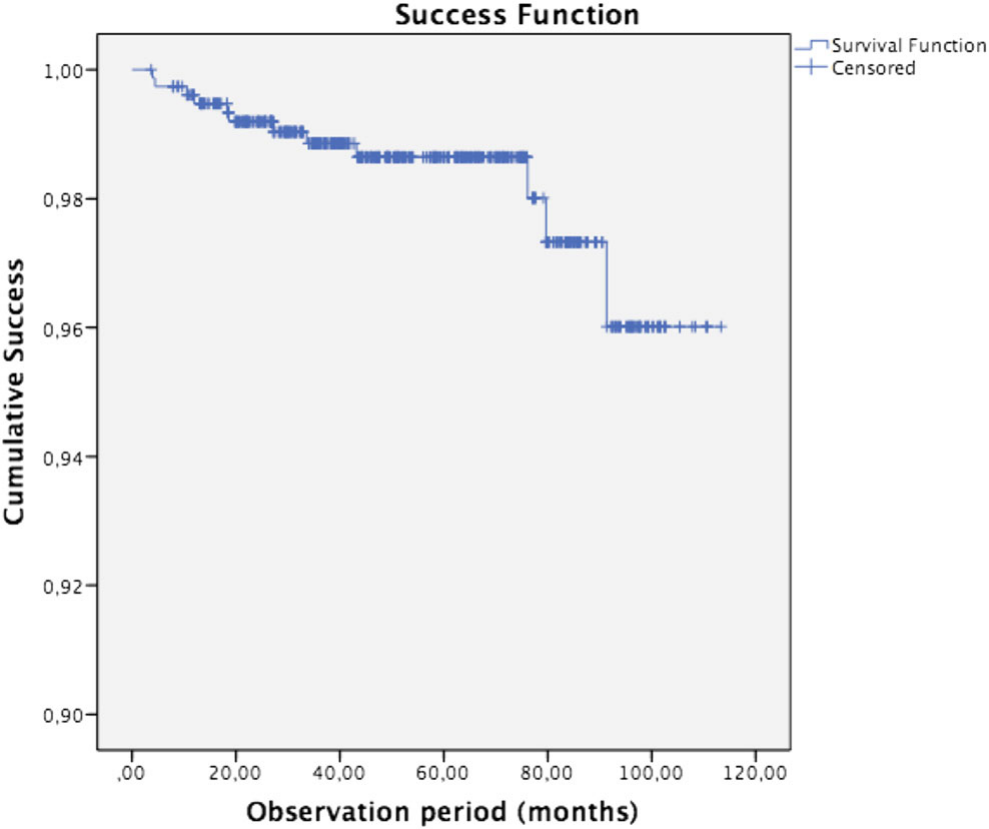
Kaplan-Meier curve of the cumulative success rate after 5 years is 98.6% SE 0.5% and 96.0% SE 1.7% after 7.5 years and longer (*n* = 765 at baseline, events *n* = 12)

The overall estimated cumulative survival rate according to Kaplan-Meier after 5 years of function and longer is 99.6% (SE 0.3%, 3 events) and the success rate are 98.6% (SE 0.5%, 9 events) after 5 years and 96% (SE 1.7%) after 7.5 years and longer (12 events, Figs. 1 and 2). Three cases were absolute failures and thus extracted, 2 being a fracture of a tooth after 10 months (devitalised) and a tooth after 33 months (vital) with restorations without cusp coverage. A non-salvageable apical re-infection occurred in another tooth after 18 months of function.

Over time, repairable and salvageable failures occurred in 9 teeth, being endodontic treatment needed (*n* = 7), secondary caries (*n* = 1) and debonding of the restoration (*n* = 1). Location (premolar/ molar and mandibular/maxilla), extension of the indirect ceramic restoration (number of sides and cusps involved) and pre-restorative endodontic treatment (vital/devitalised) did not significantly affect the cumulative success rate (log rank test, *p* > 0.05).

The USPHS data across the different observation periods are presented in Table 5 by means of frequency distributions. The condition of the vast majority of the restorations was excellent and remained unaffected after 5 years of function or longer, as also represented by the USPHS sum score. A Kruskal-Wallis test was performed to explore the USPHS sum scores in time, i.e. baseline to > 5 years. There is a statistically significant difference between the USPHS sum scores in time (*p* < 0.001). The results of the post-hoc tests after Bonferroni correction show a significant difference between the USPHS sum score at baseline and the scores for the subsequent time intervals (1, 2, 3, 4 and > 5 years). Hence, some deterioration of the general quality of the restoration is seen after the first year of clinical service, but it remained stable thereafter (Table 5).

**Table 5:** Frequency distributions (%) of the scores on the 11 USPHS criteria and USPHS sum score (mean ± SD), clustered by period in time

Photograph		Baseline	1 year	2 years	3 years	4 years	> 5 years
		(*n* = 765)	(*n* = 192)	(*n* = 166)	(*n* = 156)	(*n* = 122)	(*n* = 197)
1. Adaptation restoration (min = 0, max = 3)	0 *	100%	99.5%	99.4%	100%	99.2%	100%
	1		0.06%	0.8%			
	2						
	3						
		(*n* = 765)	(*n* = 192)	(*n* = 166)	(*n* = 156)	(*n* = 122)	(*n* = 197)
2. Caries (min = 0, max = 1)	0	100%	99%	99.4%	99.4%	100%	99.5%
	1		1%	0.6%	0.6%		0.5%
		(*n* = 765)	(*n* = 192)	(*n* = 166)	(*n* = 156)	(*n* = 122)	(*n* = 197)
3. Marginal adaptation (min = 0, max = 3)	0	100%	97.4%	97%	94.2%	96.7%	82.2%
	1		2.6%	1.8%	5.8%	3.3%	17.3%
	2			1.2%			0.5%
	3						
		(*n* = 765)	(*n* = 192)	(*n* = 166)	(*n* = 156)	(*n* = 122)	(*n* = 197)
4. Polishability (min = 0, max = 3)	0	100%	99%	97.6%	100%	100%	98.5%
	1		1%	2.4%			1.5%
	2						
	3						
		(*n* = 765)	(*n* = 192)	(*n* = 166)	(*n* = 156)	(*n* = 122)	(*n* = 197)
5. Surface staining (min = 0, max = 1)	0	100%	99%	99.4%	98.7%	99.2%	100%
	1		1%	0.6%	1.3%	0.8%	
		(*n* = 765)	(*n* = 192)	(*n* = 166)	(*n* = 156)	(*n* = 122)	(*n* = 196)
6. Contact point (min = 0, max = 1)	0	100%	99.5%	96.4%	98.1%	97.5%	99.5%
	1		0.5%	3.6%	1.9%	2.5%	0.5%
		(*n* = 765)	(*n* = 192)	(*n* = 166)	(*n* = 156)	(*n* = 122)	(*n* = 197)
7. Fracture of restoration (min = 0, max = 5)	0	100%	100%	100%	100%	100%	100%
	1						
	2						
	3						
	4						
	5						
		(*n* = 765)	(*n* = 192)	(*n* = 166)	(*n* = 156)	(*n* = 122)	(*n* = 197)
8. Wear restoration (min = 0, max = 1)	0	100%	98.4%	99.4%	99.4%	100%	100%
	1		1.6%	0.6%	0.6%		
Radiograph							
		(*n* = 765)	(*n* = 128)	(*n* = 111)	(*n* = 100)	(*n* = 109)	(*n* = 164)
9. Adaptation restoration (min = 0, max = 3)	0	100%	96.9%	100%	97%	99.1%	98.2%
	1		3.1%		3%	0.9%	1.8%
	2						
	3						
		(*n* = 765)	(*n* = 128)	(*n* = 111)	(*n* = 100)	(*n* = 109)	(*n* = 167)
10. Caries (min = 0, max = 1)	0	100%	100%	100%	100%	100%	100%
	1						
		(*n* = 765)	(*n* = 128)	(*n* = 111)	(*n* = 100)	(*n* = 109)	(*n* = 167)
11. Marginal adaptation (min = 0, max = 3)	0	100%	95.3%	98.2%	99%	98.2%	99.4%
	1		4.7%	1.8%	1%	0.9%	0.6%
	2					0.9%	
	3						
USPHS sum score (mean ± SD)**		0	1.2 ± 0.1	1.0 ± 0.0	1.0 ± 0.0	1.1 ± 0.0	1.2 ± 0.1

*Scores 0, 1, 2 and 3 can also be read as Alpha, Bravo, Charlie and Delta

**p < 0.001, post-hoc tests indicate baseline < 1 year = 2 years = 3 years = 4 years = >5 years

## Discussion

**Fig. 3:**
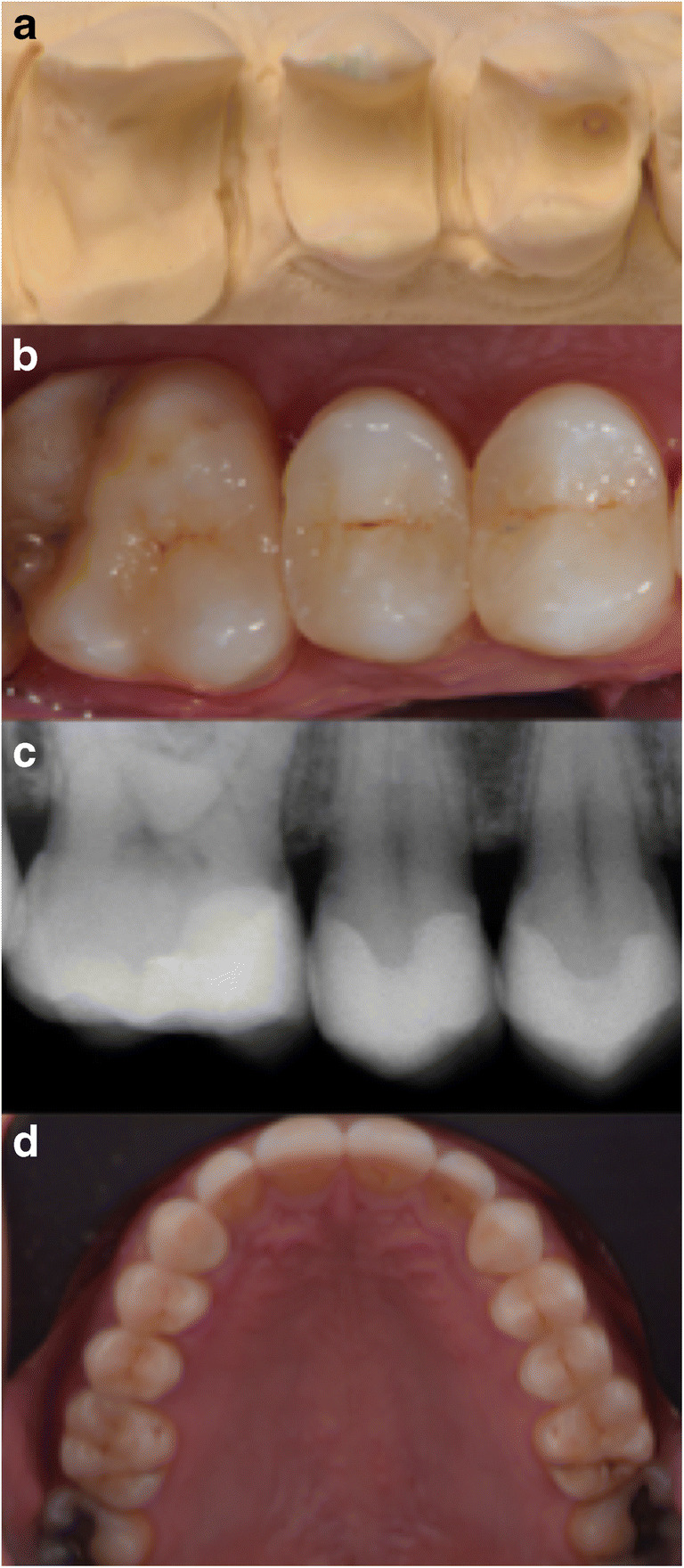
**a**–d Representative case restoring teeth 14, 15 and 16. **a** Preparation design (different case as in **b**, **c**, **d**). **b** Photograph directly after placement in 2009. **c** Radiograph directly after placement in 2009. **d** Photograph after evaluation in 2016. Total follow-up period being 102 months

In this prospective study, the clinical performance of 765 partial glass-ceramic posterior restorations luted by means of a conventional photo-polymerized resin composite in conjunction with Immediate Dentin Sealing were evaluated. Restorations presented excellent estimated cumulative survival and success rates after 5 years, being 99.6% and 98.6%, respectively. A representative case is presented in Fig. 3. No clinical study has been performed on partial ceramic restorations using Immediate Dentin Sealing and a conventional photo-polymerized resin composite.

The results of this study are better than other studies on all-ceramic partial restorations not using IDS and composite with short (2 years; 100% [32]) to medium-term (5–8 years; 94.8–97.4% [5], 7 years; 97%–100% [36],4 years; 93%–100% [37–39]) observation periods. The evidence for such restorations with longer follow-up time and high numbers of patients is limited [22].

One review reported an estimated survival rate of 95% after 5 years and 93% after 10 years [6], and compared to the survival of conventional posterior full crowns (metal–ceramic (94.7%) and lithium disilicate reinforced glass-ceramic (96.6%) after 5 years) [40], the partial restorations in this study show a higher survival rate. One of the possibilities for the excellent outcome of this study is the use of a conventional photo-polymerized resin composite as the luting agent [41]. A dual-polymerized composite resin to lute ceramic inlays was used in the vast majority of other clinical studies [5, 6, 37, 38, 42, 43]. Previous studies which investigated the use of photo-polymerized composite resins for luting purposes produced conflicting results [28, 29]. Presumed reduced wear of luting composites could not be confirmed when using the higher filled luting material [28]. However, more recent studies on this subject are promising [25–27, 31]. The higher filler content and lower initiator concentration compared to dual-polymerized resin cements may be beneficial in terms of mechanical strength and the wear properties at the exposed margins [29]. Even thick restorations are not contra-indicated with photo-polymerized luting composites in combination with IDS [25] but the use of a high power photo-polymerized (> 2000 mW/cm^2^) unit and extended polymerization time are considered of critical importance [26]. A decrease in marginal adaptation of ceramic restorations over time is reported in the current literature, as marginal deterioration can be attributed to degradation and wear of the composite [12, 44]. An important factor for the clinical long-term performance of partial coverage restorations is marginal degradation on the resin cement and deterioration of the all-ceramic materials during clinical function [45]. Based on the results of this study, the USPHS criteria according marginal adaptation showed very good results over time even with restorations up to 5 year or more because of the favourable properties of the conventional photo-polymerized resin composite.

Two absolute failures occurred due to fracture in the root of the teeth. One of these catastrophic fractures was in a vital teeth and one in a devitalised teeth. Both of these fractures occurred in teeth without cusp coverage. Several authors suggest cusp coverage to restore weak posterior teeth [46, 47]. However, the amount of 0 and 1 cusp-replaced restorations were high in this study. Almost no cusp coverage was performed due to removal of sound tissue as little as possible. The results of this study showed that the extension of the restoration and the pre-restorative endodontic status did not challenge the survival. Probably due to the adhesive quality with the use of IDS and conventional photo-polymerized resin composite, cusp coverage is no longer required. But further research is necessary to confirm this statement.

Fractures of the ceramic material or minor chipping in general were not observed, which may be contributed to meticulous polishing of the ceramic material when small occlusal corrections were deemed necessary after luting. This may have prevented micro cracks that could lead to catastrophic failures in due time [30]. An additional risk for crack formation is polymerization shrinkage of the luting composite, which creates stress concentrations at the adhesive interface and at the ceramic subsurface [48]. When using IDS with indirect bonded restorations, the delayed placement of the restorations and postponed occlusal loading facilitate the dentin bond to develop without stress [49]. The use of IDS may have led to less fractures and chippings in this study. However, the use of a lithium disilicate is also known to have higher mechanical properties that produces less fractures compared to other ceramics like leucite reinforced glass-ceramic and feldspathic glass-ceramics [1].

Chippings are reported to occur mainly at the marginal area of a restoration, involving small or severe material loss and leaving an irregular oblique fracture plane [50]. Because the condition of the restorations is difficult to assess (fracture lines and small chippings), impression taking for replicas with SEM recordings could provide additional information.

Most frequently observed failures were teeth needing endodontic treatment (1%, *n* = 7). The condition of the restorations involved with this complication was still good, with no secondary caries or defects of the restoration. From other studies, it has been reported that failures followed by endodontic complications are seen in 3% of the cases with ceramic and resin inlays, onlays and overlays [6] and in 15.6% of the cases with metal-ceramic crowns [51]. The low incidence in the presence study may be the result of the minimal invasive preparation design and the use of IDS. While a circumferential full-crown preparation is associated with the sacrifice of 67.5 to 75.6% of the original tooth structure, partial preparation is associated with substantially less sacrifice of healthy tooth tissue (5.5 to 27.2%) [52]. More invasive, circumferential crown preparations and the use of air turbines are correlated with an increase in pulpal complications [52, 53]. Exposed vital dentin immediately after tooth preparation is susceptible to insult from bacterial infiltration and micro-leakage during the provisionalization phase [13]. Bacterial and fluid penetration through the exposed dentinal tubules can result in colonization of micro-organisms, post-operative sensitivity and the potential for subsequent irritation of the pulp [13]. The use of Immediate Dentin Sealing is postulated to avoid these possible sequelae [13, 54], thereby playing an important role in keeping tooth vitality and possibly preventing teeth from hypersensitivity.

One experienced operator performed all operative procedures which increases internal, but decreases external, validity of the observations and stresses the importance of meticulous description of the operative procedures used. The difference between operators (for example seating pressure, operating time and experience) on clinical outcome is well recognized [26, 55]. However, in this study, a conventional photo-polymerized resin composite is used as a luting agent and this technique may be less technique-sensitive and therefore less prone to application errors. It is also easier to remove composite excess when a various direct composite resin rather than a low viscous material is used [26]. The use of high-quality intra-oral photographs mainly had a practical reason, so the clinical performance could be judged and critically appraised by one and the same observer, not being the treating physician, outside office times. This procedure may even be preferred over clinical evaluation when judging dental restorations [56].

The present investigation is a prospective clinical study; several clinical variables (restoration size and intra-oral distribution) could act as cofounders. Considering these limitations, further in vivo investigations would be necessary to validate the clinical performance and efficacy of lithium disilicate partial restorations, given different cementation procedures confirming the effectiveness of the approach used in this study.

## Conclusions

Under the given circumstances and conditions as presented in this prospective study, partial glass-ceramic posterior restorations luted with a conventional photo-polymerized resin composite in conjunction with the use of Immediate Dentin Sealing have an excellent medium-term prognosis.
